# Parking Slot Detection on Around-View Images Using DCNN

**DOI:** 10.3389/fnbot.2020.00046

**Published:** 2020-07-24

**Authors:** Wei Li, Hu Cao, Jiacai Liao, Jiahao Xia, Libo Cao, Alois Knoll

**Affiliations:** ^1^State Key Laboratory of Advanced Design and Manufacturing for Vehicle Body, Hunan University, Changsha, China; ^2^Chair of Robotics, Artificial Intelligence and Real-time Systems, Technische Universität München, Munich, Germany

**Keywords:** autonomous driving, parking slot detection, around-view image, DCNN, directional entrance line

## Abstract

Due to the complex visual environment and incomplete display of parking slots on around-view images, vision-based parking slot detection is a major challenge. Previous studies in this field mostly use the existing models to solve the problem, the steps of which are cumbersome. In this paper, we propose a parking slot detection method that uses directional entrance line regression and classification based on a deep convolutional neural network (DCNN) to make it robust and simple. For parking slots with different shapes and observed from different angles, we represent the parking slot as a directional entrance line. Subsequently, we design a DCNN detector to simultaneously obtain the type, position, length, and direction of the entrance line. After that, the complete parking slot can be easily inferred using the detection results and prior geometric information. To verify our method, we conduct experiments on the public ps2.0 dataset and self-annotated parking slot dataset with 2,135 images. The results show that our method not only outperforms state-of-the-art competitors with a precision rate of 99.68% and a recall rate of 99.41% on the ps2.0 dataset but also performs a satisfying generalization on the self-annotated dataset. Moreover, it achieves a real-time detection speed of 13 ms per frame on Titan Xp. By converting the parking slot into a directional entrance line, the specially designed DCNN detector can quickly and effectively detect various types of parking slots.

## 1. Introduction

With the rapid development of artificial intelligence, research on autonomous driving and driver assistance systems has drawn more and more attention from the academy and industry (Chen et al., 2020; Yurtsever et al., 2020). As a part of this, automatic parking slot detection can not only speed up the parking process and reduce traffic congestion (Paidi et al., 2018) but also assist with vehicle positioning in parking lots (Houben et al., 2019). Moreover, more and more vehicles are equipped with around-view monitor (AVM) systems to help drivers observe the surrounding road conditions. Therefore, it is of great practical meaning to detect parking slots on around-view images via existing cameras on the vehicle. However, due to illumination changes, shadows, and occlusion, it is still a big challenge to detect parking slots based on vision.

Xu et al. (2000) were the first to study vision-based parking slot detection. They detected parking slots based on the fact that the color of the parking slot markings in the image is uniform and is different from the background. However, this method is easily affected, as the values of the digital image will change greatly in different lighting scenarios. To further improve the accuracy of parking slot detection, a series of line-based methods have been proposed (Hamada et al., 2015; Lee and Seo, 2016; Lee et al., 2016). A line-based method first detects parking slot markings on the image, then clusters and fits the straight lines, and finally generates the parking space based on the geometric information of the parking slot. However, a line-based method cannot distinguish different types of parking slots, including parallel parking slots, vertical parking slots, and slanted parking slots. Different from the line-based method, Suhr and Jung (2013) proposed a marking point-based method to detect various parking slots. They first used the Harris corner detector to detect corners in the panoramic image and combined these corners into different types of junction candidates. They then matched paired junction candidates and generated parking slot candidates. Finally, a parking slot was selected based on its geometric characteristics. To improve the detection accuracy of marking points, Zhang et al. (2018) utilized a sliding window and AdaBoost classifier techniques to detect marking points of the parking slot. Li and Zhao (2018) combined line detection and marking point detection of the parking slot to further improve detection performance. However, these methods are based on low-level visual features and are not robust under complex environmental conditions.

In the last few years, DCNNs have made huge breakthroughs in different image processing tasks (Chen et al., 2019). Some methods of parking slot detection on around-view images based on DCNN have been proposed. Zhang et al. (2018) designed two DCNN models to detect parking slots, namely DeepPS. One DCNN model is based on YoloV2 (Redmon and Farhadi, 2016) for detecting marking points on around-view images. The other DCNN model is based on AlexNet (Krizhevsky et al., 2012) to match paired marking points. DeepPS has achieved good results under different environmental conditions, including indoors, outdoors, shadow, and various ground surfaces. However, DeepPS requires two DCNN models, which makes it time-consuming to infer an image. To detect parking slots with only one DCNN model, Zinelli et al. (2019) proposed an end-to-end DCNN model based on a faster region-based convolutional neural network (Faster R-CNN) (Ren et al., 2015) for parking slot detection. Since parking slots have different shapes at different viewing angles, this DCNN model directly outputs the four vertex coordinates of the parking slot instead of the bounding box aligned with the image. Li et al. (2020) utilized a YoloV3-based detector (Redmon and Farhadi, 2018) to detect parking slot heads and marking points simultaneously and then inferred the complete parking slot using the prior geometric information. However, these methods are based on the existing models, and they cannot meet the real-time requirement. To improve the speed of parking slot detection, Huang et al. (2019) proposed a directional regression method to detect parking slots, called DMPR-PS. DMPR-PS first utilizes a novel DCNN model to regress the coordinates, direction, and shape of the marking point. The geometric relationship of the parking slot is then used to match paired marking points. Although DMPR-PS improves the detection speed, it can only detect parallel parking slots or vertical parking slots.

Therefore, in order to overcome the limitations of these previous methods, we convert the problem of parking slot detection into a problem of directional entrance line regression and classification so that various kinds of parking slots can be detected quickly and robustly. Inspired by one-stage object detection methods (Liu et al., 2015; Redmon and Farhadi, 2018), we design a novel DCNN detector that can directly obtain the type, position, length, and direction of the entrance line. Based on these detection results, we can easily infer the complete parking slot using geometric information. To evaluate the performance of the proposed method, we perform several experiments on the ps2.0 dataset and a self-annotated parking slot dataset. The results show that the proposed method can efficiently detect various types of parking slots, including parallel parking slots, vertical parking slots, and slanted parking slots. The remainder of the paper is organized into three parts. Section 2 describes the method of parking slot detection based on DCNN. Section 3 presents the experimental results of our method. Section 4 presents the conclusion and discussion of this paper.

## 2. Methodology

**Figure 1:**
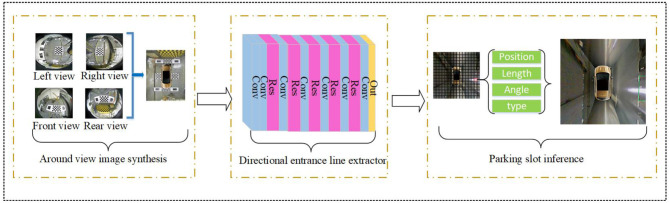
Overview of the parking slot detection method on an around-view image using a DCNN detector. It contains three modules: around-view image synthesis, directional entrance line detector, and parking slot inference.

In this section, we describe our method in detail. Figure 1 shows the overall structure of the parking slot detection method on an around-view image using a DCNN detector. Firstly, the four distortion images from the fisheye cameras are calibrated to generate an around-view image. Since the technology for AVM is very mature, we will not discuss it here and will directly use our previous research (Feng et al., 2019). Then, the around-view image is resized to the specified size as the input of the directional entrance line detector. The directional entrance line detector consists of a feature extractor and a detection head, whose details will be described in the following subsections. Finally, the complete parking slot is inferred according to the detection results and prior geometric information.

### 2.1. Structural Analysis of Parking Slots in the Around-View Image

**Figure 2:**
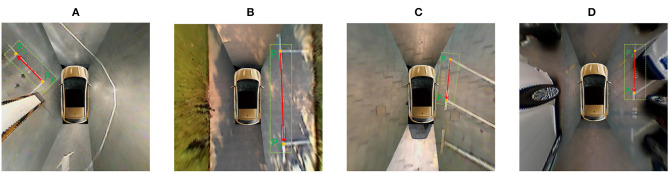
Three typical kinds of parking slots are represented. The parking slot head is marked with the green rectangle, the two visible vertices are marked with orange dots, and the entrance line is marked with the directional red line. **(A)** A vertical parking slot; **(B)** parallel parking; **(C,D)** a slanted parking slot with an acute angle and obtuse angle, respectively.

Due to the limited view of AVM, most around-view images only include the parking slot heads. As shown in Figure 2, there are three typical kinds of parking slots, which can be represented by their directional entrance lines. We stipulate that the four vertices of the parking slot are arranged counterclockwise, and the indexes of the two visible marking points closest to the vehicle are 1 and 2. Under these circumstances, the direction of the entrance line is from visible vertex *p*_1_ to visible vertex *p*_2_.

**Figure 3:**
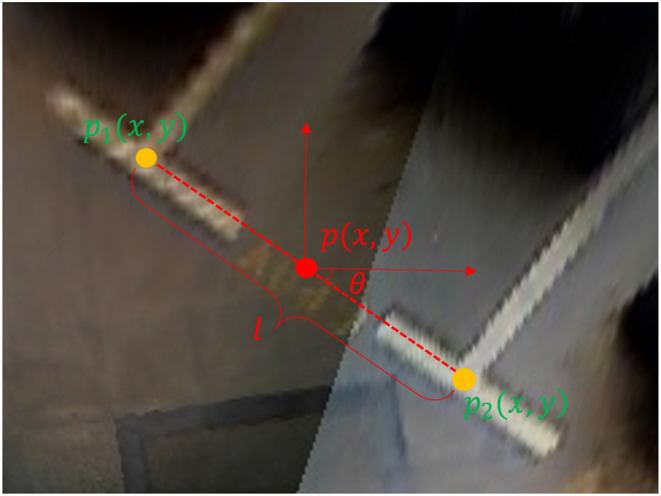
Directional entrance line representation. The entrance line is marked with a red dotted line, the two visible marking points are marked with orange dots, and the midpoint of the entrance line is marked with a red dot.

**Figure 4:**
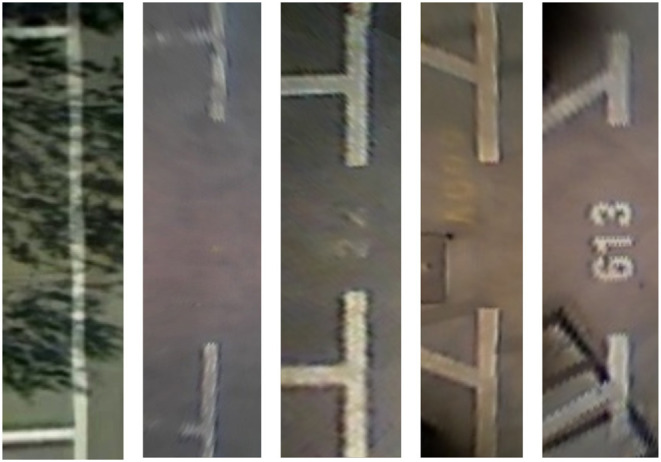
Various parking slot heads, including right-angled head, acute-angled head, and obtuse-angled head.

As shown in Figure 3, we use {*x, y*, θ, *l, c*} to represent the directional entrance line, where (*x, y*) are the coordinates of the midpoint of the entrance line, θ is the direction angle of the entrance line, and *l* is the length of the entrance line, which can be calculated by the coordinates of paired marking points of the entrance line by Equation (1). *c* represents head type, classified into a right-angled head, acute-angled head, or obtuse-angled head. As shown in Figure 4, there are different types of parking slot heads. In this way, we can convert the problem of parking slot detection into a problem of directional entrance line regression and classification.

(1) $$\begin{array}{l} \begin{array}{l} p\left(x,y\right)=\frac{p_{1}\left(x,y\right)+p_{2}\left(x,y\right)}{2\left(w,h\right)}\\ \;\theta =arctan\left(\frac{p_{2}\left(y\right)-p_{1}\left(y\right)}{p_{2}\left(x\right)-p_{1}\left(x\right)}\right)\\ \;l=\frac{\sqrt{{\left(p_{1x}-p_{2x}\right)}^{2}}+\sqrt{{\left(p_{1y}-p_{2y}\right)}^{2}}}{\lambda } \end{array} \end{array}$$

where *p*_1_(*x, y*) and *p*_2_(*x, y*) are two visible marking points of the entrance line (*w, h*), is the width and height of the input image, and λ is a normalization constant. Empirically, we chose λ = 410.

### 2.2. The Directional Entrance Line Detector

In order to make the parking slot detection efficient and effective, we specially designed a DCNN detector for directional entrance line detection. The detector mainly consists of two parts: a feature extractor and a detection head.

#### 2.2.1. Feature Extractor

**Figure 5:**
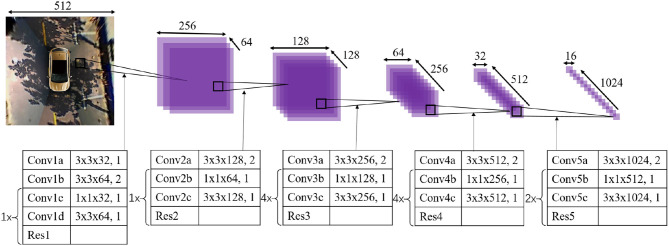
Overall structure of the feature extractor. The kernels are shown in the figures, described as height × width × depth, stride.

The overall structure of the feature extractor is based on the current object detection frameworks as well as common knowledge in this area. Considering that the task of directional entrance line detection is simpler than other tasks of target detection, we design a feature extractor with 30 convolutional layers, mainly including 3 × 3 convolution layers and 1 × 1 convolution layers. As shown in Figure 5, the input is a 512 × 512 around-view image, and the output is a 16 × 16 × 1,024 feature map. Every convolutional layer takes advantage of batch normalization and uses Leaky Rectified Linear Units (Leaky ReLU) for activation to achieve the same accuracy with fewer training steps. The shortcut connections (Res) are added for a deep network structure.

#### 2.2.2. Detection Head

The detection head is responsible for processing the feature map from the feature extractor to generate detection results. First, the feature map is passed through a 1 × 1 convolution with nine channels to output a 16 × 16 × 9 tensor; 16 × 16 represents the divided number of grids on an around-view image. Each grid has a nine-dimensional vector that includes the confidence of the entrance line *co*, the offset distance of the midpoint of the entrance line from the upper left vertex of the grid in the x-direction and y-direction (*t*_*x*_, *t*_*y*_), the length of the entrance line *l*, the cosine value *cosθ* of the direction angle of the entrance line, the sine value *sinθ* of the direction angle of the entrance line, and the class *c* of the parking slot head. Then, since the direction of the entrance line is within (−π, π), we use the Tanh function to activate for those two trigonometric values of the direction angle of the entrance line and use the Sigmod function to activate the rest. Finally, considering that two parking slots cannot overlap, we use the distance between the two midpoints of the entrance line as a judgment condition to remove duplicate parking slots.

### 2.3. Training Procedure

In the training procedure, the input is an around-view image and a label with directional entrance lines. The definition of the loss function during training is as follows.

(2) $$\begin{array}{l} L=L_{reg}\left(e,e^{*}\right)+L_{cls}\left(c,c^{*}\right) \end{array}$$

where *e* and e^*^ are the predicted value and true value of the directional entrance line, and *c* and c^*^ are the predicted class and true class of the parking slot head, which is encoded with One-Hot. The regression loss of the directional entrance line is the sum of squared errors, defined as follows.

(3) $$\begin{array}{r} \begin{array}{r} L_{reg}\left(p,p^{*}\right)=\overset{N}{\underset{i\;=\;1}{\sum }}\{{\left(co_{i}-co_{i}^{*}\right)}^{2}+\lambda \left(co_{i}=1\right)[{\left(cx_{i}-cx_{i}^{*}\right)}^{2}\\ +{\left(cy_{i}-cy_{i}^{*}\right)}^{2}+{\left(l_{i}-l_{i}^{*}\right)}^{2}+{\left(cos\theta_{i}-cos\theta_{i}^{*}\right)}^{2}\\ +{\left(sin\theta_{i}-sin\theta_{i}^{*}\right)}^{2}+\overset{2}{\underset{i\;=\;1}{\sum }}{\left(p_{i}-p_{i}^{*}\right)}^{2}]\} \end{array} \end{array}$$

where *N* is the total number of all prediction results. λ(*co*_*i*_ = 1) indicates that when the object falls into the grid *i*, it is 1, and otherwise, it is 0. *p*_*i*_ and $p_{i}^{*}$ are the predicted value and true value of the two visible vertices calculated from the directional entrance line. *p*_*i*_ can be calculated as follows.

(4) $$\begin{array}{l} \begin{array}{l} p_{1}\left(x,y\right)=p\left(x,y\right)-l\left[\begin{array}{c} cos\theta \\ sin\theta \end{array}\right]\\ p_{2}\left(x,y\right)=p\left(x,y\right)+l\left[\begin{array}{c} cos\theta \\ sin\theta \end{array}\right] \end{array} \end{array}$$

The classification loss of the head of the parking slot is the sum of binary cross-entropy loss, defined as follows.

(5) $$\begin{array}{l} L_{cls}\left(c,c^{*}\right)=\overset{N}{\underset{i\;=\;1}{\sum }}-\lambda \left(co_{i}=1\right)\left(\overset{3}{\underset{j\;=\;1}{\sum }}c_{ij}^{*}logc_{ij}-\left(1-c_{ij}^{*}\right)log\left(1-c_{ij}\right)\right) \end{array}$$

### 2.4. Parking Slot Inference

**Figure 6:**
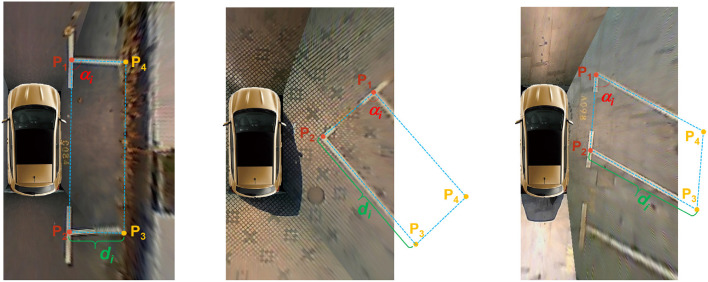
Inference of three typical parking slots. *p*_1_ and *p*_2_ are two visible vertices. *p*_3_ and *p*_4_ are two invisible vertices. *d*_1_, *d*_2_, and *d*_3_ are depth for the vertical parking slot, the parallel parking slot, and the slanted parking slot, respectively.

As shown in Figure 6, we use four vertices to represent a complete parking slot. The two visible vertices can be calculated by Equation (4) using detection results. The two invisible vertices can be calculated via Equation (6) using the angle, depth, and two visible vertices of the parking slot. The angle and depth of the parking slot can be determined according to its type. If the parking slot head is classified as a right-angle head and the distance between the two visible vertices is less than *l*_*thre*_, then it is a vertical parking slot. If the parking slot head is classified as a right-angle head and the distance between the two visible vertices is greater than *l*_*thre*_, it is a parallel parking slot. Empirically, we chose *l*_*thre*_ = 200. If the parking slot head is classified as an acute-angled head or an obtuse-angled head, it is a slanted parking slot.

(6) $$\begin{array}{l} \begin{array}{l} {\text{p}}_{3}=\left[\begin{array}{cc} \cos\alpha_{i} & -\sin\alpha_{i}\\ \sin\alpha_{i} & \cos\alpha_{i} \end{array}\right]\frac{\vec{{\text{p}}_{1}{\text{p}}_{2}}}{\left||\vec{{\text{p}}_{1}{\text{p}}_{2}}|\right|}d_{i}+{\text{p}}_{2}\\ {\text{p}}_{4}=\left[\begin{array}{cc} \cos\alpha_{i} & -\sin\alpha_{i}\\ \sin\alpha_{i} & \cos\alpha_{i} \end{array}\right]\frac{\vec{{\text{p}}_{1}{\text{p}}_{2}}}{\left||\vec{{\text{p}}_{1}{\text{p}}_{2}}|\right|}d_{i}+{\text{p}}_{1} \end{array} \end{array}$$

where α_*i*_ is the angle between the entrance line and separating line of the parking slot, and *d*_*i*_ is the depth of the parking slot. In order to obtain the prior geometric information, we perform statistical analysis on the ps2.0 dataset and a self-annotated parking slot dataset and use the average value as their true value. Therefore, we choose α_*i*_ = 90, *d*_*i*_ = 250 for the vertical parking slot, α_*i*_ = 90, *d*_*i*_ = 125 for the parallel parking slot, α_*i*_ = 67, *d*_*i*_ = 120 for the parking slot with acute-angle head, and α_*i*_ = 129, *d*_*i*_ = 120 for the slanted parking slot with obtuse-angle head.

## 3. Experiments and Results

### 3.1. Experimental Setup

#### 3.1.1. Dataset

In order to evaluate the performance of the proposed method, we conduct experiments on the following two datasets.

- **ps2.0 dataset (Zhang et al.**, **2018****):** the ps2.0 dataset is the largest around-view image dataset with parking slots, including 12,165 around-view images with 600 × 600 pixels corresponding to a ground plane of 10 × 10 m. The ps2.0 dataset is divided into a training set that contains 9,287 images and a test set that contains 2,338 images. The images in the ps2.0 dataset include indoor and outdoor scenes and three typical parking slot types. However, the labels of the ps2.0 dataset are only about the vertices of parking slots. Therefore, we need to prepare new labels for the directional entrance lines. Our DCNN detector is trained and tested on the ps2.0 dataset.
- **Self-annotated parking slot dataset:** In order to further evaluate the practical generalizability of the proposed method, we collect 2,325 images using a Peugeot 307 passenger car equipped with an AVM system that we developed earlier (Feng et al., 2019). The resolution of the image is 600 × 600 pixels, corresponding to an actual ground plane of 10 × 10 m.

#### 3.1.2. Experimental Settings

Without specifications, we implement the proposed method using Python code and the publicly available Pytorch framework in a workstation. The configuration of the workstation is as follows: Intel Core i9-7900X CPU @3.30 GHz, two Nvidia Titan Xp GPU cards, and 32 GB RAM. In the process of training, the input image is resized to 512 × 512, the batch size is 16, and the learning rate is 10^−4^. The Adam optimizer with $\left[\beta_{1},\beta_{2},\varepsilon \right]=\left[0.9,0.999,10^{-8}\right]$ is utilized to optimize the whole training process. Data augmentation is performed via adjusting brightness and contrast and adding Gaussian noise. In particular, to more accurately predict the direction of the entrance line, we have performed rotation enhancement every 5° on the entire dataset.

### 3.2. Performance of the DCNN Detector

As the first step of the proposed method, the detection performance of the directional entrance line is very important. In the experiment, we compare three detectors with different DCNNs as feature extractors, including ours, VGG16 (Simonyan and Zisserman, 2014), and ResNet50 (He et al., 2015) in the ps2.0 test set. The precision-recall curves, running time, and mean and standard deviation for the directional entrance line are used as the evaluation metrics. The precision-recall rates can be calculated as follows.

(7) $$\begin{array}{l} \begin{array}{l} precision=\frac{TruePositives}{TruePositives+FalsePositives}\\ recall=\frac{TruePositives}{TruePositives+FalseNegatives} \end{array} \end{array}$$

As defined above, for a ground truth of directional entrance line *e*_*g*_ = {*x*_*g*_, *y*_*g*_, θ_*g*_, *l*_*g*_, *c*_*g*_} and a detected directional entrance line *e*_*d*_ = {*x*_*d*_, *y*_*d*_, θ_*d*_, *l*_*d*_, *c*_*d*_}, if they satisfy the following conditions, the *e*_*d*_ is a true positive and *e*_*g*_ is correctly detected. Otherwise, the *e*_*d*_ is a false positive and *e*_*g*_ is a false negative

(8) $$\begin{array}{r} \begin{array}{rr} c_{g}=c_{d}\\ \left|l_{g}-l_{d}\right| & <10\\ \left||\left(x_{g}-x_{d},y_{g}-y_{d}\right)|\right| & <10\\ |\theta_{d}-\theta_{t}|<15\;or\;\left(360-|\theta_{d}-\theta_{t}|\right) & <15 \end{array} \end{array}$$

**Figure 7:**
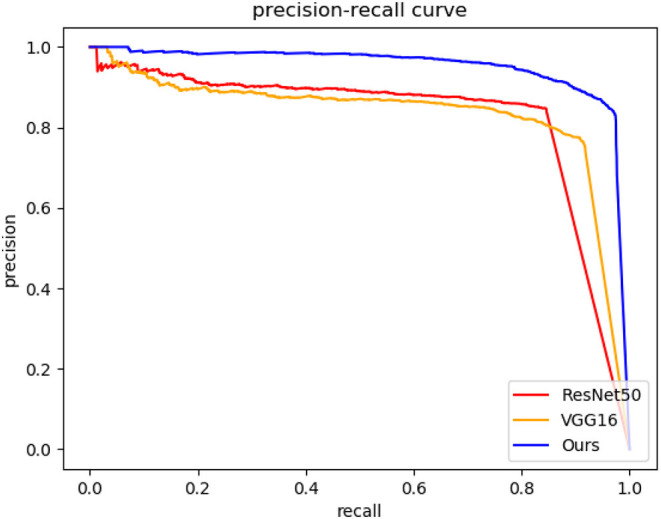
Precision-recall curves of different methods for directional entrance line detection.

The precision-recall curves of different detectors for directional entrance line detection are shown in Figure 7. The larger the enclosed area is or the higher the precision-recall curve is, the better the DCNN-based directional entrance line detection performance achieved. It is evident in Figure 7 that our method outperforms the other two detectors for directional entrance line detection. Moreover, the mean and standard deviation for the directional entrance line when the object confidence is set to 0.5 are summarized in Table 1. Compared with the VGG16-based detector and ResNet50-based detector, our method achieves higher detection accuracy with a position error of 1.49 ± 0.90, a length error of 2.26 ± 1.72, and a direction error of 3.74 ± 2.58. Although our detector has more layers than does the VGG16-based detector, ours achieves the fastest running time, 12 ms. This is because our feature extractor is composed of 3×3 and 1×1 convolution kernels, which means that it has higher computational efficiency. These results show that the specially designed DCNN detector is more suitable for directional entrance line detection than the existing networks.

**Table 1:** Directional entrance detection performance of different detectors.

**Method**	**Position**	**Length**	**Direction**	**Running time**
	**error (pixel)**	**error (pixel)**	**error (degree)**	**(ms)**
VGG16-based detector	1.68 ± 1.08	2.83 ± 2.35	4.82 ± 3.57	17
ResNet50-based detector	1.54 ± 0.97	2.63 ±2.01	4.05 ± 2.98	18
Ours	**1.49 ± 0.90**	**2.26 ± 1.72**	**3.74 ± 2.58**	**12**

*Bolded values stand for best results*.

### 3.3. Parking Slot Detection Performance

**Figure 8:**
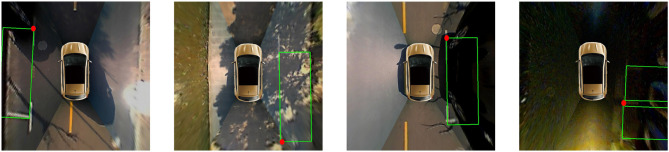
Representative images in which the parking slot is not marked in the ps2.0 test set, but our method can detect it. The detected parking slot is marked with a green box. The unclear vertex of the parking slot is marked with a red dot.

**Figure 9:**
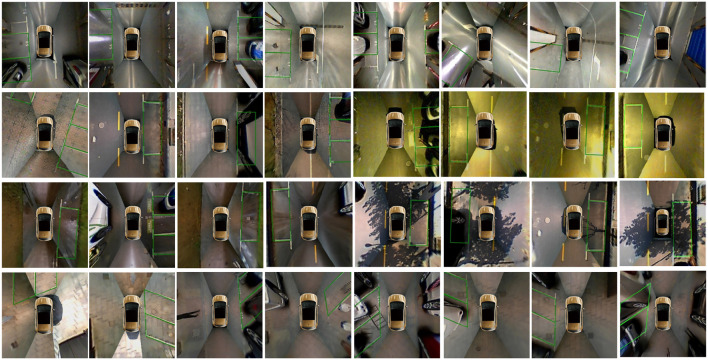
Representative parking slot detection results in the ps2.0 test set, which include various environmental conditions, such as indoor, outdoor daylight, street light, outdoor rainy, outdoor shadow, and outdoor slanted.

In this experiment, we evaluate the overall parking slot detection performance of the proposed method in the ps2.0 test set. As described in section 2.4, the parking slot consists of four vertices. If the distance between the four vertices of the detected parking slot and the four corresponding vertices of ground truth is no more than 12 pixels, respectively, it is a true positive. Otherwise, it is a false positive. In addition to our method, Table 2 also lists various SOTA methods in this field, including PSD_L (Zhang et al., 2018), DeepPS (Zhang et al., 2018), and DMPR-PS (Huang et al., 2019). It needs to be noted that when one visible vertex of the parking slot is unclear, the parking slot is not marked in the ps2.0 dataset. However, as shown in Figure 8, our method is based on directional line detection, so it can detect those parking slots even if one of their visible vertices is not clear. To compare these methods fairly, we remove these images (12 images in the ps2.0 test set). Besides, the DMPR-PS can only detect the parking slot with the right-angle head, so its precision-recall rate is evaluated on the dataset with the slanted parking slots removed. As shown in Table 2, our method gives a 1.13% higher precision rate and a 14.77% higher recall rate than PSD_L. Besides, our method also achieves a higher precision rate and recall rate than other DCNN-based methods (DeepPS and DMPR-PS). This is because PSD_L is based on machine learning (ACF + Boosting), which is easily affected by complex visual conditions, and DeepPS and DMPR-PS need complex geometric cues to match the two visible vertices of the parking slot, which might cause a mismatch. Moreover, the average time for the proposed method to process an around-view image is about 13 ms, which is almost as fast as the DMPR-PS. As shown in Figure 9, three typical kinds of parking slots under various environmental conditions can be correctly detected. These results show that the proposed method works well, and this is mainly because we convert the problem of parking slot detection into a problem of directional entrance line detection, which makes this task easier so that the designed DCNN detector can achieve detection robustly and efficiently.

**Table 2:** Parking slot detection performance of different methods in the ps2.0 test set.

**Method**	**Precision rate**	**Recall rate**	**Running time**
			**(ms)**
PSD_L (Zhang et al., 2018)	98.55%	86.64%	40
DeepPS (Zhang et al., 2018)	99.54%	98.89%	17
DMPR-PS (Huang et al., 2019)	99.42%	99.37%	**12**
Ours	**99.68**%	**99.41**%	13

*Bolded values stand for best results*.

**Figure 10:**
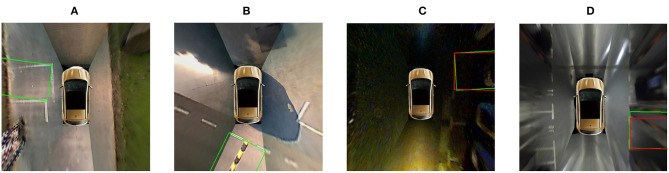
Representative failure cases of the proposed method. The detected parking slot is marked with green lines. The ground truth is marked with red lines. **(A,B)** Show the road lines being misidentified as entrance lines. **(C,D)** Show the location of the entrance lines being detected inaccurately.

However, the proposed method is still not perfect. When the parking slot is far from the vehicle, the entrance line of the parking slot is unclear, and the proposed method might miss it. Compared with false negatives, false positives are worse for automatic parking slot detection. As shown in Figure 10, four parking slots are incorrectly detected. In Figures 10A,B, the road lines are misidentified as entrance lines. In Figures 10C,D, the location of the entrance lines is detected inaccurately. In the future, we may adopt the detection results of multiple frames in the video sequence to solve this problem.

### 3.4. Generalization Performance in Practice

**Figure 11:**
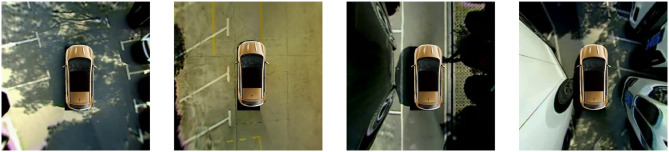
Representative images from the self-annotated parking slot dataset.

In order to further evaluate the generalizability of the proposed method, we conduct an experiment on a self-annotated parking slot dataset. The dataset was collected at Hunan University using a Peugeot 307 passenger car equipped with the AVM system that we developed earlier. Some representative images from the dataset are shown in Figure 11. The proposed method achieves a precision rate of 97.65% and a recall rate of 93.62% in the dataset. The results show that the proposed method has satisfying generalization performance.

## 4. Conclusions

In the paper, we convert the parking slot detection problem into a directional entrance line detection problem, which simplifies the learning task. Subsequently, we design a DCNN detector to detect the directional entrance line robustly. Finally, the simple geometric information of the parking slot is used to infer the complete parking slot. To evaluate the performance of the proposed method, we conduct experiments in the ps2.0 dataset and a self-annotated parking slot dataset. The results show that the proposed method not only has SOTA detection performance and satisfying generalizability in practice for various parking slots but also achieves real-time performance. However, the proposed method can still be improved in future research: (1) Fusion of multi-frame detection results in video sequences could be employed to reduce false positives and false negatives. (2) During the parking process, the entrance line is easily blocked, which leads to the parking slot being missed. Tracking of the directional entrance line can be utilized to solve this problem.

## Data Availability Statement

The datasets generated for this study are available on request to the corresponding author.

## Author Contributions

WL, HC, LC, and AK performed the conception and design of the manuscript. WL, HC, JL, and JX undertook the analysis and interpretation of data, and drafted and revised the article.

## Conflict of Interest

The authors declare that the research was conducted in the absence of any commercial or financial relationships that could be construed as a potential conflict of interest.
